# Decoding TROP2 in breast cancer: significance, clinical implications, and therapeutic advancements

**DOI:** 10.3389/fonc.2023.1292211

**Published:** 2023-10-24

**Authors:** Liqin Yao, Junfeng Chen, Wenxue Ma

**Affiliations:** ^1^ Department of Breast Surgical Oncology, The First Affiliated Hospital, Huzhou University School of Medicine, Huzhou, Zhejiang, China; ^2^ Department of Pathology and Clinical Laboratories, Tongxu County Hospital of Traditional Chinese Medicine, Kaifeng, Henan, China; ^3^ Department of Medicine, Moores Cancer Center, and Sanford Stem Cell Institute, University of California San Diego, La Jolla, CA, United States

**Keywords:** breast cancer, heterogeneity, TROP2 (Trophoblast cell-surface antigen 2), therapeutic target, clinical trials

## Abstract

Breast cancer is a heterogeneous disease characterized by distinct molecular subtypes, varied prognoses, and differential treatment responses. Understanding the molecular landscape and identifying therapeutic targets, such as trophoblast cell-surface antigen 2 (TROP2), is vital. TROP2 is notably overexpressed in breast cancer, playing a significant role in tumor growth, invasion, metastasis, and treatment resistance. While significant progress has been made in targeting TROP2 in breast cancer, several challenges and knowledge gaps remain. These challenges include the heterogeneity of TROP2 expression within breast cancer subtypes, resistance to its targeted therapies, potential off-target effects, limited therapeutic agents, and identifying optimal combination treatments. Integrating findings from clinical trials into clinical practice further complicates the landscape. This review article delves deep into TROP2 in breast cancer, highlighting its expression patterns, clinical implications, and therapeutic advancements. By understanding the role of TROP2, we can pave the way for personalized treatments, and transform the landscape of breast cancer care.

## 1 Introduction

Breast cancer remains the most commonly diagnosed cancer among women globally, accounting for approximately 30% of all new cancer diagnoses in women annually. Predictions for 2023 estimate 297,790 new invasive breast cancer cases, 55,720 new cases of ductal carcinoma *in situ* (DCIS), and an expected 43,700 breast cancer-related deaths (www.cancer.org). These statistics underlie the ongoing efforts to evolve and refine breast cancer treatment strategies.

Notably, breast cancer is a heterogeneous disease (1) with distinct molecular subtypes such as hormone receptor-positive (HR+), human epidermal growth factor receptor 2-positive (HER2+), and triple-negative breast cancer (TNBC) (2–4). Each of these subtypes exhibits unique molecular features, clinical behavior, and treatment response profiles (5, 6). Consequently, this diversity mandates tailored therapeutic strategies for effective patient outcomes.

Although progress has been made in breast cancer management, obstacles like treatment resistance and paucity of therapeutic options persist (7–9). Within this realm, trophoblast cell-surface antigen 2 (TROP2), a transmembrane glycoprotein comprising 323 amino acids, emerges as a promising candidate (10). TROP2 overexpressed is prevalent in multiple cancer types, including breast cancer, especially in the TNBC subtype (10–12). Studies have demonstrated that TROP2’s downregulation delays TNBC cell and tumor growth, underlying its oncogenic significance in breast cancer (13, 14). Furthermore, TROP2 upregulation correlates with various aggressive tumor characteristics, such as enhanced tumor growth, invasion, metastasis, and resistance to treatment (12, 15, 16).

However, translating the potential of TROP2 into effective therapeutic strategies are challenging. Heterogeneity in TROP2 expression within breast cancer subtypes can affect treatment response and clinical outcomes (17, 18). Other hurdles include resistance to TROP2-targeted therapies, potential off-target effects, and the limited arsenal of agents that specifically target TROP2 (12, 19, 20). These challenges are compounded by the intricacies of conducting clinical trials and bridging the gap between laboratory findings to clinical implementation.

It is pivotal to decode the complexities of TROP2’s role in breast cancer for progress in personalized treatment and overcoming resistance. Understanding the expression patterns of TROP2, prognostic relevance, and therapeutic innovations offers avenues for better-targeted therapies, optimizing therapeutic response, and enhancing breast cancer patient outcomes (18, 21, 22).

Despite accumulating evidence on TROP2’s therapeutic potential in breast cancer, a significant knowledge gap regarding its precise role and therapeutic application challenges (23). Addressing these gaps is essential for realizing the full promise of TROP2 as a therapeutic target in breast cancer and improving patient outcomes.

In this review, we endeavor to shed light on TROP2 in breast cancer, focusing on its expression patterns, clinical implications, and therapeutic progress. We explore the variability of TROP2 expression among different breast cancer subtypes and its correlation with clinicopathological factors. Additionally, we discuss the prognostic value of TROP2 expression, its association with treatment response, and its potential as a predictive biomarker. The latest therapeutic innovations targeting TROP2, including monoclonal antibodies (mAbs), antibody-drug conjugates (ADCs), and immunotherapeutics, are also examined. Finally, our focus shifts to prospective avenues and challenges in harnessing TROP2 therapeutically. Emphasizing the necessity for more in-depth research to elucidate TROP2’s molecular mechanisms and navigate the obstacles in developing effective TROP2-targeted therapies, we aim to uplift patient outcomes and reshape breast cancer treatment paradigms.

## 2 Molecular landscape and therapeutic targeting strategies in breast cancer

### 2.1 Overview of breast cancer subtypes

The heterogeneity of breast cancer manifests as distinct molecular subtypes, each with specific molecular characteristics and clinical behaviors. These subtypes significantly influence treatment approaches and outcomes. The major subtypes include HR+, HER2+, and TNBC, each has its own therapeutic considerations (6, 24).

In a recent groundbreaking multi-omics study conducted by Jin et al. (25), the intricate molecular landscape of breast cancer, with a specific focus on the HR+/HER2- subtype, was underscored, shedding light on its profound implications for therapeutic responses and outcomes. This study unveiled an immunogenic subtype enriched with immune cells, signifying the potential benefits of immunotherapy for this specific breast cancer subtype.

Therapeutic considerations are different for each subtype. HR+ breast cancers, primarily driven by estrogen receptor (ER) and/or progesterone receptor (PR) are amenable to endocrine therapies. These receptors serve as therapeutic targets, and as such, endocrine therapies are highly effective in this subtype (26). Hormone-based treatments, such as selective estrogen receptor modulators and aromatase inhibitors, play a pivotal role in managing HR+ breast cancers. Similarly, HER2+ breast cancers are targetable with HER2-directed therapies. Targeted therapies, including HER2-directed monoclonal antibodies like trastuzumab and pertuzumab, have revolutionized the treatment of HER2+ breast cancers (27). These targeted treatments specifically inhibit HER2 signaling, leading to improved outcomes. In contrast, TNBC, which lacks ER, PR, and HER2 expression, presents a formidable challenge in treatment due to the absence of precisely targeted therapies. Current approaches for TNBC include conventional chemotherapy and ongoing research into novel therapies, including immunotherapy and targeted agents (28).

In summary, the treatment landscape for breast cancer is significantly influenced by the specific molecular subtype, with each subtype requiring distinct therapeutic strategies. Understanding the molecular intricacies of breast cancer, particularly the HR+/HER2- subtype, is crucial for optimizing therapeutic responses and outcomes, including the potential benefits of immunotherapy, as emphasized in the recent multi-omics study by Jin et al. (25).

### 2.2 Therapeutic targets in breast cancer

Though traditional treatments such as surgery, chemotherapy, radiation therapy, and hormonal therapy have undoubtedly improved outcomes for many breast cancer patients. However, the persisting challenges such as treatment resistance (28–30), and disease recurrence (31), necessitate the identification of novel therapeutic targets. By homing in on the molecular driver of tumor progression, targeted therapies promise precision and efficacy, minimizing treatment-related toxicities (32–34).

### 2.3 TROP2 as a potential target

TROP2, a 323 amino acids transmembrane glycoprotein (10), is prominently overexpressed in various epithelial cancer types, including breast cancer, especially the TNBC subtype (10–12). Its oncogenic attributes in breast cancer, such as driving tumor growth and progression, have been documented (13, 14). Elevated TROP2 expression is linked with aggressive tumor characteristics, including enhanced tumor growth and metastasis (12, 15, 16), making it a promising therapeutic target. Furthermore, while TROP2’s expression in normal tissues is subdued (35), its pronounced expression in breast cancer presents a potential therapeutic window (14, 36).

### 2.4 Role of TROP2 in breast cancer progression

**Figure 1:**
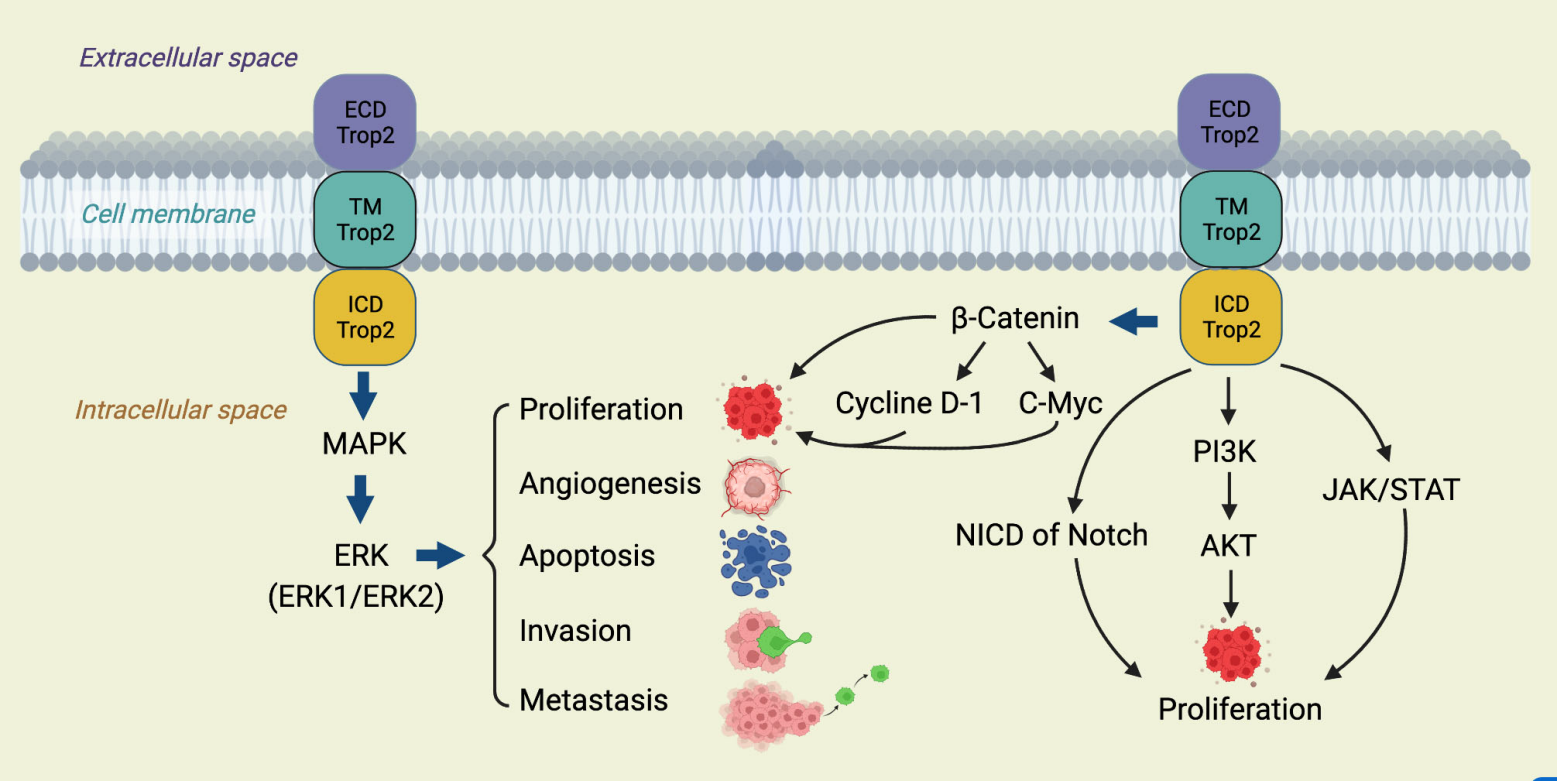
TROP2’s central role in tumorigenic signaling pathways.

TROP2’s involvement in breast cancer progression spans various facets, from promoting cell proliferation to resisting therapies (12). Crucially, it activates several tumorigenic signaling pathways, like the Wnt/β-catenin and EGFR-linked MAPK/ERK and PI3K/Akt pathways (12, 37). Furthermore, its influence extends to matrix metalloproteinases, which facilitate cancer cell invasion (12, 38), and it also plays a role in maintaining CSCs, known for their association with tumor recurrence and therapy resistance (39). The multifaceted roles of TROP2, as elucidated through these pathways, underscore its potential as a therapeutic target (**Figure 1**).

This diagram illustrates the multifaceted involvement of TROP2 in various oncogenic signaling cascades. TROP 2 activates the ERK1/2-MAPK axis, promoting malignant transformation and driving tumorigenesis. Additionally, it modulates the Notch pathway, influencing stem cell functions and potential tumor differentiation and hierarchy (40). TROP2 also interacts directly with nuclear β-catenin, propelling cell proliferation, a hallmark of cancer (41). A comprehensive understanding of these pathways, as depicted, offers insights into potential therapeutic targets in TROP2-driven cancers.

## 3 Expression patterns and clinical significance of TROP2 in breast cancer

### 3.1 Heterogeneity of TROP2 expression within breast cancer subtypes

TROP2 exhibits notable overexpression in TNBC, a subtype distinguished by its aggressive phenotype, establishing it as a pivotal therapeutic target and prognosis biomarker (18, 36). Conversely, TROP2 overexpression in HR+ breast cancer is more subdued but prominently pronounced in HER2+ breast cancer, suggesting a potential avenue for combination therapies targeting both TROP2 and HER2 (13, 18, 42). Additionally, luminal B (ER+, or PR-, HER2-) breast cancer, known for its less favorable prognosis relative to luminal A (ER+, PR+, HER2-), also displays significant TROP2 overexpression (22). Understanding the variability of TROP2 expression across subtypes is imperative for tailoring treatments effectively.

### 3.2 Clinical implications of TROP2 expression

Elevated TROP2 expression levels in breast cancer correlate with unfavorable prognostic markers, including larger tumor size, and increased risk of recurrence (43, 44). Specifically, in TNBC, heightened TROP2 levels are linked to increased tumor aggression and resistance to chemotherapy (45–47). Furthermore, TROP2’s potential as a therapeutic target is highlighted in HER2+ breast cancer, where combined treatment modalities may enhance outcomes (18, 36, 42). The integration of TROP2 expression analysis into treatment decisions holds promise for optimizing therapeutic strategies.

### 3.3 Correlation between TROP2 expression and clinicopathological factors

Studies on the associations between TROP2 expression and clinical-pathological characteristics in TNBC present mixed findings. While some studies found no substantial correlation between TROP2 levels and clinicopathological factors in breast cancer, like age, histologic subtype, tumor grade, stage, lymphovascular invasion, or tumor-infiltrating lymphocytes (TILs) levels (36), the significance of TROP2 as a prognostic factor in breast cancer remains undeniable. Further investigations in this domain could refine personalized treatment strategies.

### 3.4 TROP2 as a predictive biomarker

TROP2 stands out as a promising predictive biomarker in breast cancer, with its expression levels informing on treatment response. Elevated TROP2 expression levels in tumors have been associated with resistance to specific therapies, such as chemotherapy and endocrine treatments (48). Notably, in the realm of targeted therapies, TROP2-expression has shown potential in enhancing responsiveness to drugs like Sacituzumab Govitecan in specific breast cancer subtypes (48). However, mechanisms of resistance, potentially linked to the upregulation of multidrug resistance proteins, are an area warranting further investigation. The intricate relationship between TROP-2 and other cellular pathways emphasizes the need for a comprehensive approach to leveraging its potential as a therapeutic target.

In summary, TROP2’s expression patterns and implications in breast cancer solidify its stature as both a prognostic and predictive biomarker. As we advance our understanding, the insights gathered can guide clinical decisions, promote personalized treatments, and improve patient outcomes.

## 4 TROP2-targeted therapies in breast cancer clinical trials

Numerous clinical trials are currently investigating TROP2-targeted therapy for breast cancer, including mAbs, ADCs, and CAR T-cell therapies. These therapies hold great promise for advancing breast cancer treatment by targeting TROP2 specifically and effectively.

### 4.1 mAbs and ADCs targeting TROP2

Several TROP2-targeted mAbs are under evaluation, with a focus on improving therapeutic efficiency while sparing healthy cells (49, 50). Notably, Liu et al. reported promising results with T-cell-redirecting bispecific antibodies (TRBAs) targeting TROP2 and CD3, which suppressed tumor growth in both TNBC cell lines and primary tumor cells (51).

Promising ADCs under investigation include PF-06664178 (46, 52), IMMU-132 (46, 53, 54), and DS-1062a (46, 55). These agents target TROP2-expressing cancer cells and are under clinical evaluation for their therapeutic potential in TROP2-positive breast cancer.

#### 4.1.1 PF-06664178

Developed by Pfizer, PF-06664178 represents a cutting-edge ADC drug that utilizes a humanized IgG1 mAb targeting TROP2, a prominent antigen on breast cancer cells. The mechanism of this ADC is intriguing: once it binds to TROP 2 and is internalized by the cancer cell, it is directed to the lysosomes, it is within these cellular compartments that the ADC releases its cytotoxic payload, the auristatin-based compound known as Aur0101 (38). Preliminary studies investigating PF-06664178 have yielded encouraging outcomes. These initial findings depict a drug with modest antitumor activity, which has sparked significant interest in the scientific and medical communities. As a result, more comprehensive evaluations and clinical trials are now underway to determine its therapeutic potential and safety profile in treating TROP2-positive breast cancer patients (52).

#### 4.1.2 Sacituzumab Govitecan

Sacituzumab Govitecan (Trodelvy or IMMU-132) is an FDA-approved ADC tailed for TROP2-positive cancers, particularly TNBC. Its mAb component specifically targets TROP2, delivering the cytotoxic agent SN-38 directly to the tumor cells. Clinical trials have demonstrated its effectiveness for metastatic TNBC patients, positioning it as a promising frontline treatment (56–58).

#### 4.1.3 Datopotamab deruxtecan

Dato-DXd, or DS-1062a, represents a promising addition to the landscape of TROP2-targeted therapies in breast cancer. This innovative ADC pairs a TROP2-targeting antibody with topoisomerase I inhibitor payload, creating a highly specific and effective therapeutic approach (57). The rationale behind Dato-DXd lies in its dual mechanism of action. The TROP2-targeting antibody ensures precise binding to TROP2-expressing breast cancer cells, delivering the therapeutic payload with pinpoint accuracy (59). The attached topoisomerase I inhibitor disrupts the cancer cell’s DNA replication and repair processes, leading to cell death. Ongoing clinical trials are examining its potential for treating advanced breast cancer (37).

#### 4.1.4 SKB264

SKB264, also known as AKB264, represents another noteworthy member of the TROP2-targeted ADC family. It shares the same mAb as IMMU-132, which targets the TROP2 receptor in breast cancer cells, is being investigated for its potential therapeutic effects. Current clinical trials aim to ascertain its effectiveness in various breast cancer stages, particularly in advanced forms (49). The key feature of SKB264 lies in its potential to harness the specificity of the TROP2-targeting antibody, ensuring precise binding to TROP2-expressing breast cancer cells (60).

### 4.2 CAR T-cell therapy

CAR T-cell therapy is emerging as a promising approach for TROP2-positive cancers, including breast cancer. Chen et al. developed a CAR targeting Trop2 (T2-CAR) with different co-stimulatory intercellular domains and found that T2-CAR T cells exhibited robust cytotoxic activity against Trop2-positive cells *in vitro*. Moreover, these T2-CAR T cells produced a plethora of effector cytokines upon antigen stimulation (61). Interestingly, when a CD27 intercellular domain was incorporated, the antitumor activity of T2-CAR T cells was enhanced, especially in tumor-bearing mouse models. These CD27-based T2-CAR T cells demonstrated a higher survival rate in the spleens and tumor tissues of tumor-bearing mice and exhibited upregulated IL-7Rα expression and downregulated PD-1 expression, indicating a multifaceted mechanism of enhanced killing effect.

In another study, Zhu et al. demonstrated that CAR T-cells equipped with a fully human single-chain variable fragment (scFv) targeting TROP2 effectively killed TROP2-positive pancreatic cancer cells and inhibited tumor growth in xenograft models. These findings suggest that TROP2-CAR T-cells, including breast cancer, can be a potent therapeutic strategy for TROP2-positive cancer types. In another study, Zhao et al. developed bi-specific CAR T-cells targeting TROP2 and PD-L1 and showcased their superior tumoricidal activity in both *in vitro* and *in vivo* settings (62). Collectively, these results indicate the potential of bi-specific CAR T-cells as an emerging immunotherapeutic strategy for TROP2-positive cancers, including breast cancer. As CAR T-cell therapy continues to evolve, further research and clinical investigations are crucial to realize its full therapeutic potential.

In conclusion, TROP2-targeted therapies are gaining momentum as potential treatments for breast cancer. Ongoing clinical trials will continue to define the role of these therapies in the treatment landscape. For a comprehensive list of ongoing clinical trials focusing on TROP2 inhibitors in TNBC, please refer to **Table 1**.

**Table 1:** Current recruiting clinical trials involving TROP2 inhibitors in TNBC.

Study Title	Interventions	Phase	Study Design	Number Enrolled	NCT Number	Primary Completion
A Study of ZEN003694 and Talazoparib in Patients with Triple Negative Breast Cancer	ZEN003694, Talazoparib	Phase 2	Allocation: Non-Randomized, Intervention Model: Parallel Assignment, Masking: None (Open Label), Primary Purpose: Treatment	179	NCT 03901469	November 2023
Avelumab With Binimetinib, Sacituzumab Govitecan, or Liposomal Doxorubicin in Treating Patients with Stage IV or Unresectable, Recurrent Triple Negative Breast Cancer	Anti-OX40 Antibody PF-04518600, Avelumab, Binimetinib (and 3 more…)	Phase 2	Allocation: Randomized, Intervention Model: Parallel Assignment, Masking: None (Open Label), Primary Purpose: Treatment	150	NCT 03971409	June 30, 2024
First-in-human Study of DS-1062a for Advanced Solid Tumors (TROPION-PanTumor01)	Datopotamab Deruxtecan (Dato-DXd), Steroid Containing Mouthwash, Non-Steroid Containing Mouthwash	Phase 1	Allocation: Randomized, Intervention Model: Sequential Assignment, Masking: None (Open Label), Primary Purpose: Treatment	890	NCT 03401385	January 1, 2025
A Study of Dato-DXd With or Without Durvalumab Versus Investigator**’**s Choice of Therapy in Patients with Stage I-III Triple-negative Breast Cancer Without Pathological Complete Response Following Neoadjuvant Therapy (TROPION-Breast03)	Dato-DXd, Durvalumab, Capecitabine, Pembrolizumab	Phase 3	Allocation: Randomized, Intervention Model: Parallel Assignment, Masking: None (Open Label), Primary Purpose: Treatment	1075	NCT 05629585	September 20, 2027
A Study of Dato-DXd Versus Investigator**’**s Choice Chemotherapy in Patients with Locally Recurrent Inoperable or Metastatic Triple-negative Breast Cancer, Who Are Not Candidates for PD-1/PD-L1 Inhibitor Therapy (TROPION-Breast02)	Dato-DXd, Paclitaxel, Nab-paclitaxel (and 3 more…)	Phase 3	Allocation: Randomized, Intervention Model: Parallel Assignment, Masking: None (Open Label), Primary Purpose: Treatment	600	NCT 05374512	December 3, 2025
Fudan University Shanghai Cancer Center Breast Cancer Precision Platform Series Study- Neoadjuvant Therapy	Dalpiciclib, Pyrotinib, SHR-A1811 (and 13 more…)	Phase 1 Phase 2	Allocation: Randomized, Intervention Model: Parallel Assignment, Masking: None (Open Label), Primary Purpose: Treatment	716	NCT 05582499	September 2024
Sacituzumab Govitecan in Primary HER2-negative Breast Cancer	Capecitabine, Carboplatin, Cisplatin, Sacituzumab govitecan	Phase 3	Allocation: Randomized, Intervention Model: Parallel Assignment, Masking: None (Open Label), Primary Purpose: Treatment	1332	NCT 04595565	March 30, 2027

## 5 Prospects and challenges in TROP2-targeted therapies

The future of breast cancer treatment sees promise in advanced interventions such as ADCs, next-generation CAR T-cell therapies, and novel immunotherapeutic interventions. Integrating TROP2-targeted therapies with conventional treatments could enhance efficacy and patient outcomes. Crucial steps ahead include biomarker validation, understanding resistance mechanisms, and refining therapeutic avenues through rigorous clinical trials and translational research. Integrating TROP2-targeted therapy into personalized medicine and ensuring equitable access are important objectives to enhance patient care.

Nevertheless, targeting TROP2 in breast cancer treatment is not devoid of challenges. Ensuring therapy specificity is vital to minimize toxicity in normal tissues. The varied nature of breast cancer subtypes necessitates strategies capable of addressing each subtype effectively. Key challenges lie in surmounting treatment resistance, pinpointing predictive biomarkers, and gaining an in-depth understanding of TROP2 signaling dynamics. Addressing these aspects will pave the way for fully harnessing the potential of TROP2-targeted therapy in breast cancer treatment.

## 6 Concluding remarks

TROP2’s role in breast cancer, highlighted by its pronounced overexpression and pivotal function in tumor dynamics, establishes it as a compelling therapeutic target. With an array of promising TROP2-centric interventions, from mAbs, ADCs, to CAR T-cell therapy, the landscape of treatment, especially for TNBC, is evolving. The endorsement of Sacituzumab Govitecan by the FDA for metastatic TNBC highlights this potential. However, the journey is not without obstacles, with resistance emergence, of breast cancer subtypes variation, and the need for reliable biomarkers being foremost. To truly harness the promise of TROP2-based interventions, these challenges mandate focused research. The horizon of TROP2-targeted strategies shines brightly with promise for advancing breast cancer therapeutics.

## Author contributions

LY: Conceptualization, Project administration, Resources, Supervision, Validation, Visualization, Writing – review & editing, Methodology, Writing – original draft. JC: Data curation, Formal Analysis, Investigation, Methodology, Resources, Validation, Writing – original draft, Writing – review & editing. WM: Conceptualization, Formal Analysis, Investigation, Project administration, Resources, Supervision, Validation, Visualization, Writing – review & editing.

## Glossary

ACS: American Cancer Society
ADC: Antibody-drug conjugate
CAR: Chimeric antigen receptor
CSCs: Cancer stem cells
DCIS: Ductal carcinoma in situ
DFS: Disease-free survival
ECD: Extracellular domain
EGFR: Epidermal growth factor receptor
ER: Estrogen receptor
ERK: Extracellular signal-regulated kinase
EMT: Epithelial to mesenchymal transition
HER2: Human epidermal growth factor receptor 2
HR: Hormone receptor
mAbs: Monoclonal antibodies
MAPK: Mitogen-activated protein kinase
NICD: Intracellular domain of the notch protein
MMP: Matrix metalloproteinase
OS: Overall survival
PI3K: Phosphoinositide 3-kinases
PR: Progesterone receptor
scFv: Single-chain variable fragment
SIT: Short intracellular tail
STAT: Signal transducers and activators of transcription
TD: Transmembrane domain
TNBC: Triple-negative breast cancer
TROP2: Trophoblast cell-surface antigen 2
